# [Retracted] Long non-coding RNA SNHG3 promotes the development of non-small cell lung cancer via the miR-1343-3p/NFIX pathway

**DOI:** 10.3892/ijmm.2024.5421

**Published:** 2024-09-09

**Authors:** Lijun Zhao, Xue Song, Yesong Guo, Naixin Ding, Tingting Wang, Lei Huang

Int J Mol Med 48: 147, 2021; DOI: 10.3892/ijmm.2021.4980

Following the publication of this paper, it was drawn to the Editors' attention by a concerned reader that certain of the Transwell cell migration and invasion assay data shown in Fig. 3B were strikingly similar to data appearing in different form in a pair of other articles written by different authors at different research institutes, one of which had already been published elsewhere prior to the submission of this paper to *International Journal of Molecular Medicine*, and one of which was under consideration for publication at around the same time. In view of the fact that the abovementioned data had already apparently been published previously, the Editor of *International Journal of Molecular Medicine* has decided that this paper should be retracted from the Journal. The authors were asked for an explanation to account for these concerns, but the Editorial Office did not receive a satisfactory reply. The Editor apologizes to the readership for any inconvenience caused.

